# Monthly Summary

**Published:** 1872-03

**Authors:** 


### MONTHLY SUMMARY.

*Oxygen Gas in Pulmonary Diseases.*—Dr. H. M. Read reports in the *N. Y. Medical Journal* a series of cases of lung disease, in which the inhalation of oxygen diluted with common air was added to other treatment. In some instances great benefit accrued, and in others none. Experiments similar in kind were tried in England by Dr. Beddoes and others nearly a century ago. The doctor was very sanguine that he had discovered at last a cure for many cases of consumption. But for some reason the practice was abandoned, to be revived now as a novelty.—*Pacific Med. and Surg. Jour.*

*Carbon Bisulphide, Rhigolene, and Oleum Menthæ Piperitæ as Local Anæsthetics.*—Dr. S. R. Nissley, of Pemberton, Ohio, writes:—"I have been in the habit of using the bisulphide of carbon as a local anæsthetic for several years. I have tested its efficacy and patency in facial neuralgia, hemicrania, odontalgia, and lumbago, and the relief it afforded to the sufferer was almost instantaneous. My mode of application was this: place a pledget of cotton into a wide-mouthed bottle, saturate it well with the bisulphide and apply it to the painful part, and as soon as the patient complains of smarting sensation change the bottle, carefully following the course of the principle nerve that seems to be involved in the difficulty. I have used a combination of rhigolene and the oil of peppermint as a local anæsthetic in a number of neuralgic cases that presented themselves at my office for relief, and thus far my success in those cases has been far beyond my most sanguine expectations. After several applications, they express themselves cured. I have recently been in the habit of adding an etherial collodion to the compound, and I am gratified to say that in the combination I have a specific which will, under almost any circumstance, when the part is accessible, relieve the patient instantaneously. Its effects are magical. In a severe case of tic douloureux this compound was tried, and the patient was relieved.—*Jour. Mat. Med.*

---

*Chloride of Zinc with Chloride of Potassium.*—Ordinary chloride of zinc is unsatisfactory as an escharotic, because of its hygroscopic tendency. M. Bruns states that this defect may be overcome by fusing two parts of the chloride of zinc with one of the chloride of potassium, and casting in sticks—the latter to be wrapped in tin foil.—*Practitioner and Pharmacist.*

---

*Burns and Scalds.*—Every family should have a preparation of flaxseed oil, chalk, and vinegar, about the consistency of thick paint, constantly on hand for burns and scalds. A noted retired physician states that he has used it in hospital and private pratice for the past forty years, and believes that no application can compare with it, as regards relief of pain and curative results.—*Druggists' Circular.*

